# Corrigendum: Enhancing oncolytic virotherapy by extracellular vesicle mediated microRNA reprogramming of the tumour microenvironment

**DOI:** 10.3389/fimmu.2025.1601201

**Published:** 2025-04-22

**Authors:** Victoria A. Jennings, Reah Rumbold-Hall, Gemma Migneco, Tyler Barr, Katrina Reilly, Nicola Ingram, Isabelle St Hilare, Samuel Heaton, Noura Alzamel, David Jackson, Christy Ralph, Susan Banerjee, Iain McNeish, John C. Bell, Alan A. Melcher, Carolina Ilkow, Graham P. Cook, Fiona Errington-Mais

**Affiliations:** ^1^ Leeds Institute of Medical Research, School of Medicine, University of Leeds, St. James University Hospital, Leeds, United Kingdom; ^2^ Center for Innovative Cancer Therapeutics, Ottawa Hospital Research Institute, Ottawa, ON, Canada; ^3^ Leeds Cancer Centre, St James’s Hospital, Leeds, United Kingdom; ^4^ The Royal Marsden Hospital, Fulham Road, London, United Kingdom; ^5^ Department of Cancer and Surgery, Imperial College London, London, United Kingdom; ^6^ The Institute of Cancer Research, Divisions of Radiotherapy and Imaging and Breast Cancer Research, Chester Beatty Laboratories, London, United Kingdom

**Keywords:** oncolytic virus, miRNA, tumour associated macrophage, tumour microenvironment, anti-tumour immunity

In the published article, there was an error in the article title. Instead of “Enhancing oncolytic virotherapy by extracellular vesicle mediated microRNA reprograming of the tumour microenvironment”, it should be “Enhancing oncolytic virotherapy by extracellular vesicle mediated microRNA reprogramming of the tumour microenvironment”.

In the published article, there was an error in the **Funding** statement. Several funders were omitted from the original statement. The correct **Funding** statement appears below.

“The authors declare that financial support was received for the research, authorship, and/or publication of this article. Ovarian Cancer Action funded Victoria Jennings, Reah Rumbold-Hall and the costs required to undertake this study. Alan Melcher was supported by CRUK (Programme grant CRM183X), Tyler Barr was supported by the Myrovlytis Trust and by an MRC/NIHR Cancer Research Transatlantic Development and Skills Enhancement Award (NIHR304246), Samuel Heaton by an MRC Discovery Medicine North (DiMeN) studentship (MR/N013840/1) and Noura Alzamel by a scholarship from Kuwait University. Nicola Ingram is supported in part by the National Institute for Health and Care Research (NIHR) Leeds Biomedical Research Centre (BRC) (NIHR213331). Other authors were supported by their respective institutions. The views expressed are those of the author(s) and not necessarily those of the NHS, the NIHR or the Department of Health and Social Care, or any other funders of this work.”

In the published article, there was an error in the **Acknowledgements**. Funders others than Ovarian Cancer Action were omitted from the original statement. The correct statement appears below.

“The authors would like to extend their sincere gratitude to Ovarian Cancer Action for their generous funding and support, which made this research possible. Their commitment to advancing ovarian cancer research, has been instrumental in enabling us to carry out this study. We thank the other organisations who supported the study and thank our colleagues Robert Salmond and Sandra Bell for the kind gift of materials and for their scientific input into this study.”

The authors apologize for this error and state that this does not change the scientific conclusions of the article in any way. The original article has been updated.

