# Supplementary figures and images for: Enhancing oncolytic virotherapy by extracellular vesicle mediated microRNA reprogramming of the tumour microenvironment
Source: Front Immunol. 2024 Dec 23;15:1500570. doi: 10.3389/fimmu.2024.1500570 (PMC11701023; doi:10.3389/fimmu.2024.1500570)

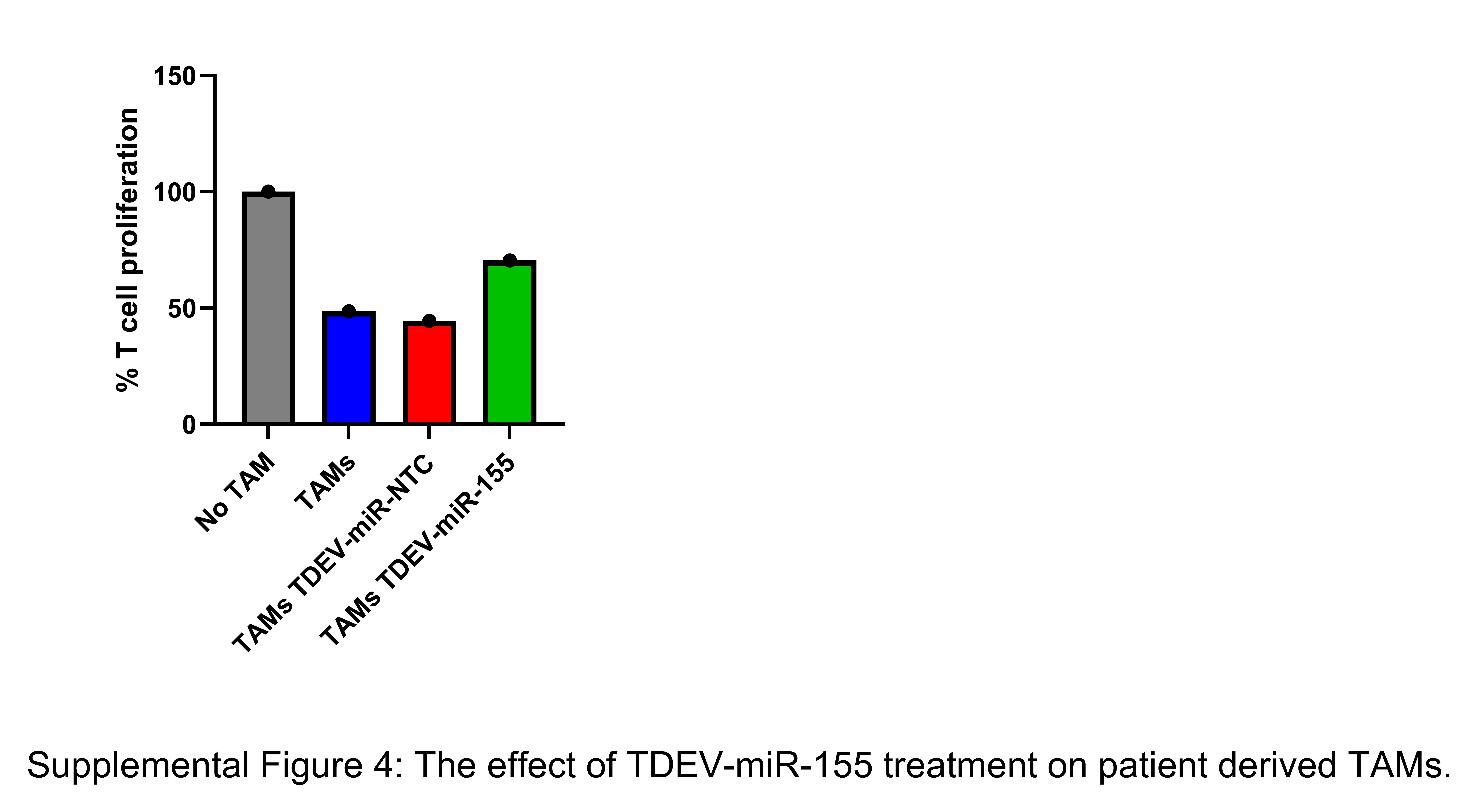

Supplement: Supplementary Figure 4 — The effect of TDEV-miR-155 treatment on patient derived TAMs. Percentage of T cell proliferation (via tritiated thymidine incorporation) following five days stimulation with CD3/CD28 Dynabeads cultured with, no TAMs, ascitic fluid isolated TAMs either co-cultured with TDEV-miR-NTC or TDEV-miR-155 overnight prior to T cell co-culture (n=1). [file Image4.jpg]
